# Application of botulinum toxin to treat sialorrhea in amyotrophic lateral sclerosis patients: a literature review

**DOI:** 10.1590/S1679-45082016RB3594

**Published:** 2016

**Authors:** Ademar Francisco de Oliveira, Gêssyca Adryene de Menezes Silva, Débora Milenna Xavier Almeida

**Affiliations:** 1 Associação Caruaruense de Ensino Superior e Técnico, Faculdade Asces, Caruaru, PE, Brazil

**Keywords:** Sialorrhea, Amyotrophic lateral sclerosis, Salivary glands, Botulinum toxins, Ultrasonography

## Abstract

Amyotrophic lateral sclerosis is a progressive and fatal neurodegenerative disease characterized by the degeneration of motor neurons, which are the central nervous system cells that control voluntary muscle movements. The excessive salivation (sialorrhea) is present in approximately 50% of amyotrophic lateral sclerosis cases. Thus, some alternative therapeutic methods are sought, such as anticholinergic drugs and surgery. Recently the use of botulinum toxin applied at a midpoint of the salivary glands, often guided by ultrasound, have demonstrated positive results. The objective was to review the literature to demonstrate an alternative method to treatments of sialorrhea in patients with amyotrophic lateral sclerosis. In recent studies, the efficacy of botulinum toxin is confirmed, although new applications are required. Since the side effects are negligible, this is an alternative to treat amyotrophic lateral sclerosis, and other patients with diseases that present sialorrhea.

## INTRODUCTION

Amyotrophic lateral sclerosis (ALS) is a fatal progressive neurodegenerative disease, characterized by secondary muscle weakness and involvement of motor neurons, which are the central nervous system cells that control voluntary muscle movement. Two types of motor neurons are affected by ALS: upper motor neurons (UMN), or first neurons, which are located in the motor area of the brain; and lower motor neurons (LMN), or second neurons, which are located in the brainstem and in the anterior portion of the spinal cord.

The UMNs regulate LMN activity through chemical messages (neurotransmitters). LMN activation allows the contraction of the body's voluntary muscles and, in the brainstem, LMNs activate muscles in the face, mouth, throat, and tongue. The LMNs, in the spinal cord, activate all other voluntary muscles of the body, such as those of upper and lower limbs, trunk, neck, and diaphragm, among others.^(1)^

The ALS action in LMN muscles may generate excessive salivation (sialorrhea). Costa et al.,^(2)^ demonstrated the clinical classification of sialorrhea as mild (exteriorizing saliva in small quantities, mild stasis, with low risk of pulmonary aspiration); moderate (exteriorizing saliva in moderate quantities; use of up to three bibs per day); and severe (large exteriorization of saliva, use of more than three bibs per day, laryngeal penetration of saliva, and high risk of pulmonary aspiration).

The bacterium *Clostridium botulinum* produces a toxin that causes botulism. Toxins produced by *C. botulinum* are among the most lethal substances known in science.^(1,2)^ There are seven different types of known toxins that can be produced by *C. botulinum,* designated by the letters: A, B, C, D, E and F. Although the toxins present structural similarities, their action sites are different, resulting in variable effects. Only type A has been approved for clinical use in Brazil. Recently, with a greater effect on autonomic junctions, type B use was approved in the United States (Myobloc, Elan, San Francisco, CA, USA).

Certain toxins produced by the botulism-causing bacteria may be purified and industrialized. Thus, this toxin of poisonous nature takes on the name botulinum toxin Botox^®^, used mainly for the reduction of wrinkles and facial expression lines.^(3–5)^ Recently, Botox^®^ has been used not only for aesthetics purposes, but also as treatment for some symptoms of degenerative diseases, such as ALS and Parkinson's disease, among others.

Botox^®^ administration in the salivary glands of patients with certain neurological disorders aims to decrease sialorrhea caused by failure in facial and oral muscle control that acts during swallowing, and aids phonation parameters and facilitates inclusion of the patient in social contexts.^(6,7)^ Several studies were done with the objective of demonstrating the alternative method that aims to aid in the treatment of sialorrhea, one of the symptoms of neurodegenerative disease patients. Among these studies, we highlight Portes,^(3)^ who assessed ten patients between 5 and 21 years of age, all over 10kg and dysphagic; Costa et al.,^(2)^ who developed research with 22 patients who had botulinum toxin administered in their salivary glands [14 (63.3%) males, and 8 (36.4%) females, age range 4 – 34 years, with a mean of 12.5 years and a median of 13 years]; and Manrique,^(8)^ who selected five ALS patients diagnosed at least two years prior for ultrasound-guided injection of Botox^®^ in salivary glands. In total, 37 patients were evaluated, 35 (94.6%) of which had ALS, and 2 (5.4%) with other neurodegenerative conditions (Chart 1).

**Chart 1:** Summary of the main studies that used botulinum toxin in amyotrophic lateral sclerosis patients

Author	Study	Participants	Results
Costa CC, et al.^(2)^	Injections of botulinum toxin into the salivary glands to the treatment of cronic sialorrhoea.	22	Reduction of sialorrhea in all cases. Two patients presented little reduction due to the presence of multicystic glands. No side-effects.
Portes KP^(3)^	*Aplicação da toxina botulínica em glândulas salivares como tratamento da sialorréia crônica em pacientes com doença neurológica.*	10	Clear improvement in the picture of all patients, with reduction in the number of daily bib changes. No side effects.
Manrique D^(8)^	Application of type A botulinum toxin to reduce saliva in amyotrophic sclerosis lateral.	5	Improvement in 4 of 5 patients, with no side effects. Three patients went 4 months with no complaints.

### Sialorrhea: conventional treatments or use of botulinum toxin?

Healthy individuals secrete between 1,000 to 1,500mL of saliva in 24 hours. Many neurologic diseases evolve in what concerns excessive salivation, presenting the patient with difficulties in oral motor control.^(7)^ Manrique^(8)^ stated that when saliva production exceeds the individual's ability to transport it from the mouth to the stomach, stasis, drooling and aspiration may occur, as well as concomitant difficulties in mastication and articulation. Sialorrhea occurs in approximately 50% of ALS patients, 70% of Parkinson's disease patients, and between 10 and 80% of patients with cerebral palsy.^(8)^

Sialorrhea in patients with ALS hampers mastication and speech, with possible drooling and difficulty in salivary aspiration, directly affecting the patient's quality of life in the social context, hindering his/her inclusion and worsening depression, as well as also making rehabilitation more difficult.^(8,9)^ Other proposals aiming at saliva reduction are already routinely used, such as anticholinergic drugs that cause some side effects, including urine retention and headache.^(9,10)^

For patients at a not very advanced stage of the disease, surgical approach is a possibility, consisting of removal of one of the salivary glands. It is a delicate procedure that exposes the patient to general anesthesia risks.

Thus, alternative therapeutics are required. Botox^®^ steps away from the field of aesthetics to be used as palliative treatment for neurodegenerative diseases. For its injection, the patient needs to go through dental treatment beforehand and should not have used Botox^®^ in other sites in the previous 6 months.

Botox^®^ treatment is still not the first option. The administration of botulinum toxin is performed upon intolerance to anticholinergic effects. Some salivary glands, such as the submandibular gland, are not easily accessed through palpation, thus requiring ultrasonography guidance.^(11)^

### Ultrasonography-guided type A Botulinum toxin application

The submandibular gland is slightly difficult to be detected by palpation only.^(8)^ Hence, we should rely on imaging resources, such as ultrasonography, through which we can detect the gland and inject the toxin in hard-to-reach glands, such as the submandibular.^(12–14)^ Ultrasound guidance is recommended to locate salivary glands. Mean doses range between 20 to 30 units for each of the parotid gland, and 10U for the submandibular gland. Effect duration varies between 2 and 6 months, and longer durations of the effect are related to doses that are also greater; however, in these cases, adverse reactions may occur, such as dry mouth and dysphagia.^(14,15)^ This is a little invasive procedure and, although it demands a new application after a few months, it does not present side effects, unless the administered amount is superior to the cited volume, or the patient has any type of anaphylaxis to botulinum toxin. Some studies reported the administration of 30U of Botox^®^ in a central point of the salivary gland with no side effects or systemic effects. In 10% (4 cases), Botox^®^ action was not present, whereas in 90% (33 cases), there was improvement of salivation, which was prolonged by continued application.^(8,15)^

Botox^®^ administration is presented as the best alternative in relation to more invasive procedures, such as surgery to excise one of the glands. The use of anticholinergic drugs may bring several types of side effects, such as urinary retention and headache. Treatment with Botox^®^ has shown positive results in the studies done so far. Still, there is a *deficit* in the quantity of works published in this context, which suggests the need for more research and articles to demonstrate this practice and make it more popular.

**Figure 1:**
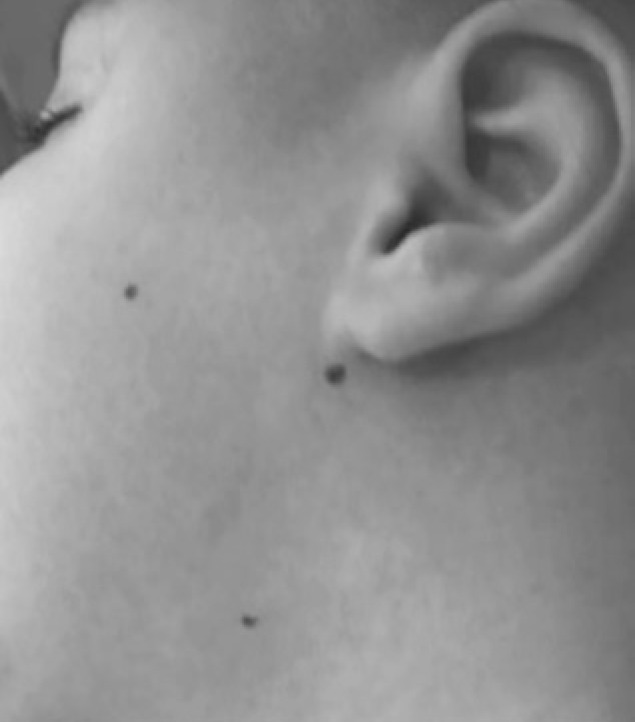
Botulinum toxin application points in salivary glands. Parotid gland (two points) and submandibular glands (one point)

Source: Manrique D. [Application of type A botulinum toxin to reduce saliva in amyotrophic sclerosis lateral]. Rev Bras Otorrinolaringol. 2005;71(5):566-9. Portuguese.^(8)^

**Figure 2:**
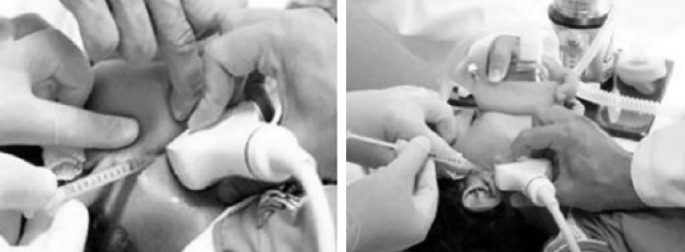
Direct ultrasound-guided botulinum toxin application in the parotid and submandibular glands, under sedation

Source: Costa CC, Ferreira JB. [Injections of botulinum toxin into the salivary glands to the treatment of cronic sialorrhoea]. Rev Bras Cir Cabeça Pescoço. 2008;37(1):28-31. Portuguese.^(2)^

Botulinum toxin broadens horizons and brings the possibility of greater benefits with lower risks, even if it requires periodical applications. Local or systemic side effects are not manifested if the application does not surpass the recommended volume of up to 30U in the parotid glands and 10U in the submandibular glands, according to the points in figure 1, which leads us to believe that, if an effect arises, it will be of anaphylactic nature. Despite all advancements of modern science, there is currently no cure for ALS, but there are palliative treatments. The use of botulinum toxin has demonstrated positive results when administered to patients with chronic sialorrhea, affected by neurodegenerative diseases, assuming there are no complications regarding the employed technique (Figure 2). The possibility of generating side effects is extremely small, considering they can only be of anaphylactic nature. Sialorrhea control provides better feeding and social interaction, reducing embarrassment and enhancing the patient's self-esteem.

Out of 37 patients studied, 32 (86.5%) had ALS and one (2.7%) had Parkinson's disease, and 33 (90%) improved their clinical picture. There was significant reduction in the number of bibs used, from four to two or three per day. Patients who used up to three bibs per day stopped using it. Those who presented severe sialorrhea moved to the moderate group, and those who were moderate moved to the mild group. Four patients (10.8%) did not present any improvement; in that, 3 (8.1%) had ALS and 1 (2.7%), Parkinson's disease (Table 1). In some patients with multicystic salivary glands, the results were inferior to those of patients without these pathological alterations.^(8,16)^

**Table 1:** Results of botulinum toxin studies in patients with amyotrophic lateral sclerosis and Parkinson's disease

Related disease	Improvement n (%)	No improvement n (%)
Amyotrophic lateral sclerosis	32 (86.5)	3 (8.1)
Parkinson's disease	1 (2.7)	1 (2.7)

## CONCLUSION

Surgical procedures and anticholinergic drugs, among other methods, have been used many times to control sialorrhea. Nowadays, science has broadened its horizons, bringing an alternative aesthetic procedure to aid in the well-being of neurodegenerative disease patients who manifest the symptom of excessive salivation in their clinical condition, improving the patient's life in the physical, psychological and social aspects.
